# Implementing quality management strategies improves clinical quality as a voluntary medical male circumcision program in Namibia matures: a process analysis

**DOI:** 10.1186/s12913-023-10016-6

**Published:** 2023-09-29

**Authors:** Gillian O’Bryan, Alison Ensminger, Idel Billah, Edwin Sithole, Magdaleena Nghatanga, Laura Brandt, Mark Shepard, Mekondjo Aupokolo, Assegid Tassew Mengistu, Norbert Forster, Brigitte Zemburuka, Gram Mutandi, Scott Barnhart, Gabrielle O’Malley, Caryl Feldacker

**Affiliations:** 1 International Training and Education Center for Health (I-TECH), Seattle, WA USA; 2 Department of Global Health, University of Washington, Seattle, WA USA; 3 International Training and Education Center for Health (I-TECH), Windhoek, Namibia; 4 Centers for Disease Control and Prevention (CDC/DDPHSIS/CGH/DGHT), Windhoek, Namibia; 5 Directorate of Special Programmes-Ministry of Health and Social Services, Windhoek, Namibia; 6 Department of Medicine, University of Washington, Seattle, WA USA

**Keywords:** Voluntary Medical Male Circumcision (VMMC), Namibia, Adverse events, Quality, HIV prevention

## Abstract

**Background:**

Surgical voluntary medical male circumcision (VMMC) is a safe procedure; however, maintaining quality standards at scale, particularly during scale-up, is a challenge making ongoing quality management (QM) efforts essential. This study describes program quality measured by rates of adverse events (AEs) over four years of VMMC implementation in Namibia, compares AE rates over time, and discusses QM processes that contextualize AE trends and illustrate improvements in quality as the program matured. The International Training and Education Center for Health (I-TECH) assisted the Namibian Ministry of Health and Social Services (MoHSS) in expanding VMMC in three regions among boys and men over 10 years of age between January 2015 and September 2019.

**Methods:**

A comprehensive package of QM strategies was implemented by multi-disciplinary onsite teams with support from national and international technical advisors. Retrospective routine MoHSS data from the VMMC register, client forms, and monthly AE reports were collected during implementation in the three regions to assess the impact of QM interventions on AEs and to calculate the proportion of clients who experienced AEs over time. The proportion of clients who experienced an AE over time was compared using a Cochran-Armitage test for trend.

**Results:**

Between January 2015 and September 2019, 40,336 clients underwent VMMC and 593 (1.5%) clients experienced a post-operative AE in the three supported regions. The AE rate was highest in the first quarter of clinical service delivery in each region (January-March 2015 in Oshana and Zambezi, October-December 2017 in //Kharas) but declined over the implementation period as the program matured. This observed trend between program maturity and declining AE rates over time was significant (p < 0.001) when compared using a Cochran-Armitage test for trend.

**Conclusions:**

As the I-TECH-supported VMMC program matured, QM measures were introduced and routinized, and clinical quality improved over time with the rate of AEs decreasing significantly over the implementation period. Applying systematic and continuous QM processes and approaches across the continuum of VMMC services and considering local context can contribute to increased clinical safety. QM measures that are established in more mature program sites can be quickly adopted to respond to quality issues in program expansion sites.

## Background

Voluntary medical male circumcision (VMMC) programs have been rapidly scaled, particularly in Southern and Eastern Africa, to meet World Health Organization (WHO) and Joint United Nations Programme on HIV and AIDS (UNAIDS) goals of 5 million VMMCs performed annually and the U.S. President’s Emergency Plan for AIDS Relief (PEPFAR) targets of 80% circumcision coverage of the eligible population [1, 2].

When implemented in line with internationally established guidance, VMMC is a safe procedure [3]. To maintain the safety of the procedure, it is essential to achieve and sustain quality standards, particularly during rapid scale-up [4]. Early studies of VMMC programs in sub-Saharan Africa have raised questions regarding the extent to which health systems can deliver VMMC safely according to minimum quality standards at scale [4]. Concerns are that programs implemented without meeting quality standards can result in surgical complications stemming from inadequate provider competence and training, poor clinical infrastructure, limited patient information, and incomplete follow-up [4, 5]. Additional published data on the quality of mature, large-scale VMMC programs are urgently needed to ensure lessons learned from safe programs are shared and newly established programs can adopt promising practices from inception.

Quality management (QM) comprises all activities designed to achieve and sustain high-quality outputs and encompasses quality assurance (QA), quality control (QC), and quality improvement (QI) approaches [6, 7]. QA and QC focus on compliance with minimum predefined, measurable quality standards, and monitoring of performance within existing process designs [8, 9]. In contrast, QI identifies gaps in quality and focuses on continuously reviewing and redesigning processes to achieve improved levels of performance while monitoring results [8, 9]. QM assesses the quality of care provided and informs and measures changes to maintain or improve service delivery quality. Three key components of QM are defining, measuring, and improving quality. Successful QM approaches and processes must consider local context to ensure responsiveness to the implementing environment [5, 10].

The WHO defined strategies for safeguarding VMMC quality, also incorporated into PEPFAR-funded program guidance, emphasize ensuring provider competence, monitoring and evaluating program outcomes including adverse events (AEs), and developing and implementing standard operating procedures and best practices for providing information and education to clients and communities [5, 11].

Rates of AEs, especially during rapid program scale-up, are a key indicator of VMMC program quality given the critical importance of patient safety and good clinical outcomes in large scale VMMC efforts [12]. Adverse events include injury, harm or any undesired outcome that occurred during or following the VMMC procedure and comprise of three components: (1) type (bleeding, pain, infection, swelling, wound disruption, scar or disfigurement, injury to glans, excessive skin removal); (2) severity (mild, moderate, severe); and (3) timing (intraoperative, postoperative) [12]. AE rates in clinical trials, active surveillance settings, and VMMC programmatic implementation range from 0.5 to 8% [13–30]. Rates below 2% are internationally recognized as acceptable-levels for moderate and severe AEs combined [12]. A reduction in AE rate, or sustainment of a low rate at scale, can indicate clinical quality [12].

The objectives of this analysis are to (1) describe program quality measured by the rate of AEs that occurred over four years of program implementation in Namibia; (2) describe trends in AE rates over time; and (3) describe implemented QM processes to provide context to observed trends in AEs within Namibia and illustrate improvements in program quality as the VMMC program matured. This analysis builds on previously published work that provided a summary of the overall AE profile of Namibia’s VMMC program including AE type and timing, association of VMMC characteristics and AEs, as well as factors associated with AE severity [29].

## Methods

### VMMC program

In 2010, the Namibian Ministry of Health and Social Services (MoHSS) launched its National Policy on Male Circumcision for HIV Prevention [31]. To help accelerate the roll-out of the policy, the International Training and Education Center for Health (I-TECH) was engaged by the U.S. Centers for Disease Control and Prevention (CDC) and MoHSS to provide technical assistance and direct service delivery support for VMMC in three regions with high HIV prevalence, low VMMC coverage, and not practicing traditional circumcision between January 2015 and September 2019. In January 2015, I-TECH began direct service delivery of VMMC in the Oshana and Zambezi regions in the north of Namibia and in October 2017 was requested to expand to the //Kharas region in southern Namibia. This study only includes data from surgical VMMC as device-based circumcision had not yet been approved by the MoHSS.

The VMMC program was staffed by multi-disciplinary teams consisting of clinical, demand creation, and strategic information staff at the site level. The teams operated a main VMMC clinic integrated into the MoHSS hospital in each district, as well as regular outreach services at community-based primary healthcare (PHC) clinics and at other community institutions (e.g., schools, prisons). All VMMC clinical staff completed theoretical and proficiency training including required completion of 20 VMMC procedures under direct supervision of an experienced and accredited supervisor prior to certification. The three regional site-teams were supported by a strategic leadership team based in Windhoek, Namibia, as well as Technical Advisor, Monitoring and Evaluation Advisor, and Senior Program Manager based in Seattle, USA. The Windhoek-based technical team provided close supervision and support to the site teams and undertook regular site visits that included onsite training, mentoring, and coaching on the implementation of QM measures. This multi-disciplinary team approach facilitated an environment in which service quality was prioritized, challenges were continuously identified, and contextually driven solutions were implemented.

### Quality management measures

**Fig. 1:**
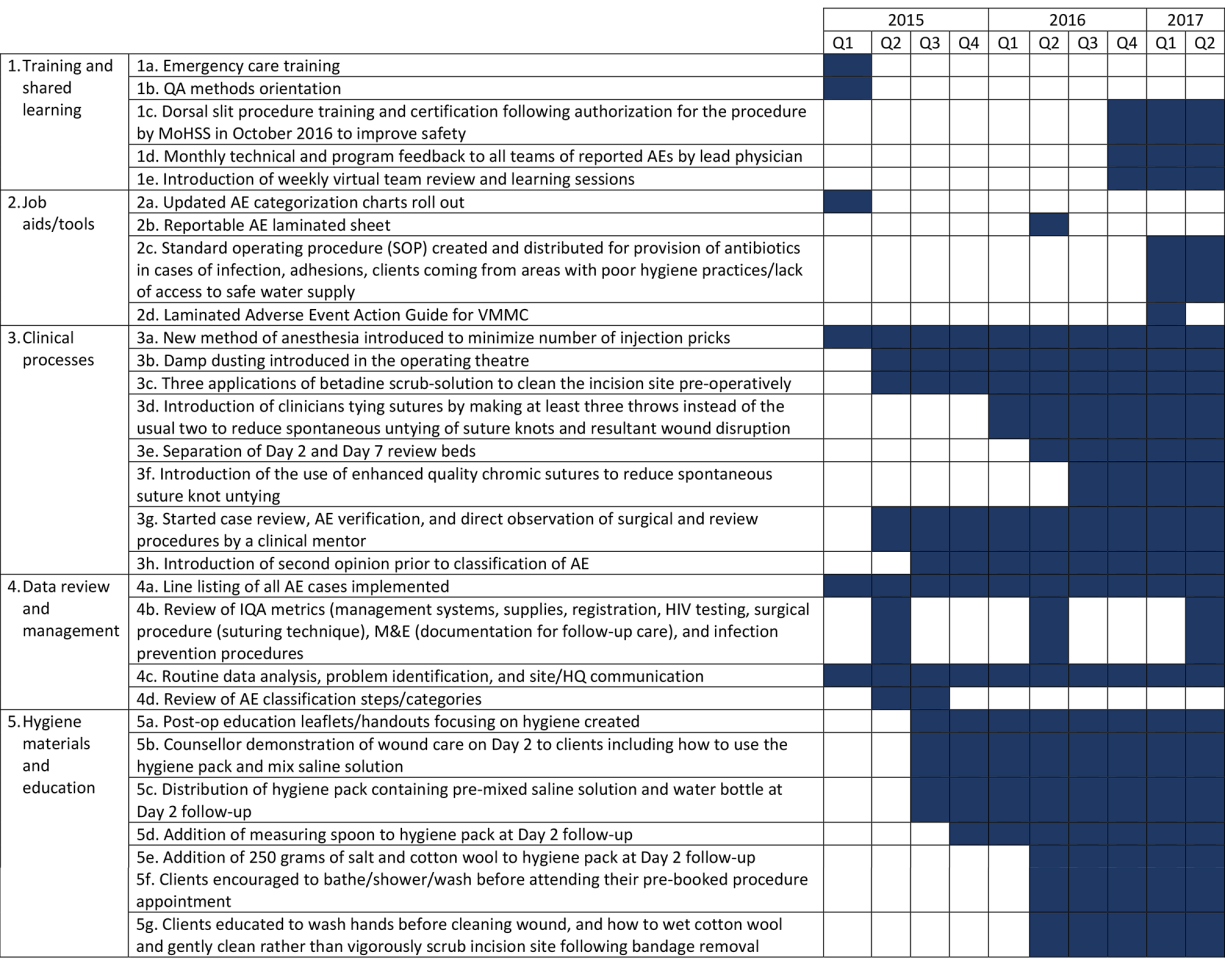
VMMC AE QM interventions in the Oshana, Zambezi, and //Kharas regions of Namibia (January 2015-Mach 2017)

The program implemented a comprehensive package of QM measures described in Fig. 1 between January 2015 and March 2017. Figure 1 focuses on early program implementation when QM measures were introduced through March 2017. All QM strategies outlined in Fig. 1 were implemented in the Oshana and Zambezi regions and later implemented as part of program expansion in the //Kharas region in October 2017. Several background processes facilitated QM interventions including the introduction of internal quality assessments (IQA) and external quality assessments (EQA), and establishment of QM teams and formalized AE review groups. Upon program start up in February 2015, Oshana and Zambezi site teams conducted a baseline IQA. A subsequent IQA was conducted in 2016 and in 2017 transitioned to an EQA conducted under the leadership of the MoHSS. Between October and November 2015, QM teams were established in Oshana and Zambezi. These QM teams were responsible for monitoring, making recommendations, implementing activities to ensure clinical quality standards were met or exceeded, and further reinforcing a culture of continuous quality improvement. Finally, in December 2015, AE review committees were formed to assess all reported AEs monthly through establishment of a line list containing key information about each AE case for review and discussion with technical experts.

Overall, the QM processes were implemented across three intervention levels: clinician facing interventions, client and community facing interventions, and interventions targeting program systems and processes. Clinician-facing activities safeguarded VMMC provider competency through training, development and implementation of job aides, and tools for continuous improvement of clinical service delivery. Client and community-facing activities focused on patient and caregiver education related to hygiene, pain management, and post-operative wound care, as well as provision of targeted wound care supplies and counseling. Systems-level QM measures focused on data review and management through implementation of new tools, new or modified clinical procedures, review of assessment and monitoring processes, and promotion of routine team-based data use to improve quality. This was supported by virtual weekly progress review and learning sessions which brought together site-level and above site-level team members.

### Ethics

This study was conducted under a routine data use protocol approved by the U.S. Centers for Disease Control and Prevention (CDC) and the MoHSS. and received a non-research determination from the University of Washington institutional review board. In Namibia, adults < 18 years must obtain written approval from a parent or guardian for VMMC surgical procedures, no additional verbal or written consent from patients was not sought for this secondary analysis of the de-identified, aggregate data.

### Data collection

For this analysis, we compiled routine monthly MoHSS AE reports (January 2015–September 2019) of individuals with moderate and severe AEs kept by clinicians at each site. MoHSS monthly program summary reports compiled from site-level VMMC registers and client forms by I-TECH’s site-level staff were used to establish the denominator of the number of VMMCs performed. The number of VMMCs performed served as the denominator in the calculation of AE rate as follow-up completion was nearly 100%. Both the AE line list data and the summary report data were analyzed at an aggregate level and disaggregated by location and program year.

Internal program documents such as site visit reports, IQA and EQA reports, and emails and meeting notes were reviewed to identify QM activities implemented during the study period and to detail illustrative vignettes that demonstrate how quality issues were identified and resolved.

### Data analysis

The proportion of clients who experienced AEs were calculated using the compiled routine program data of the total clients who underwent VMMC during the implementation period as well as the total clients who experienced an AE (as identified by the line lists). The proportion of clients who experienced an AE over time was compared using a Cochran-Armitage test for trend. Confidence intervals (CI) (95% percentile) for AE rates by site calculated using Wilson estimates for trend analysis. This analysis was conducted using STATA 16.

### Definition of terms

Two surgical circumcision techniques were used during the study period. Forceps-guided and dorsal slit VMMC were approved for clients ages 15 and older; for clients ages 10 through 14, only the dorsal slit VMMC procedure was approved. Clinicians determined the surgical type for clients aged 15 and above based on glans maturity. In Namibia, VMMC for clients aged 10–14 formed part of the 2010 Namibia National VMMC Policy and the dorsal slit procedure was authorized through an official policy circular issued by the MoHSS in October 2016.

Generally, severe AEs require surgical intervention or hospitalization. Any AE not classified as severe but requiring medical intervention was considered moderate [11, 12]. Mild AEs were not reported nor included in this analysis.

The level of program maturity is represented by program duration.

## Results

During the study period, 40,336 VMMCs were performed in three regions with 593 clients documented in the line lists as experiencing an AE (Table 1). Over the implementation period, the average monthly AE rate for both moderate and severe AEs was 1.5%, with a range of 0–5.2% per month as reported in monthly monitoring data. By site, the AE rate ranged from 0 to 5.7% in Zambezi, 0-5.9% in Oshana, and 0.2-11.5% in //Kharas.

**Table 1:** Adverse events related to VMMC procedures in three I-TECH supported regions of Namibia (January 2015–September 2019): //Kharas, Oshana, and Zambezi

		Total VMMC Clients	Total AEs n (%)
Year			
	Year 1 (Jan-Sep 2015)	6,251	207 (3.31%)
	Year 2 (Oct 2015-Sep 2016)	7,686	166 (2.16%)
	Year 3 (Oct 2016-Sep 2017)	8,004	89 (1.11%)
	Year 4 (Oct 2017-Sep 2018)	9,140	76 (0.83%)
	Year 5 (Oct 2018-Sep 2019)	9,255	55 (0.59%)
Age*			
	10–14	13,492	92 (0.68%)
	15–19	12,486	193 (1.55%)
	20+	14,358	303 (2.11%)
Region			
	Oshana	22,011	294 (1.34%)
	Zambezi	14,562	253 (1.74%)
	//Kharas	3,763	46 (1.22%)
Season			
	Oct-Dec	4,270	70 (1.64%)
	Jan-Mar	5,706	108 (1.89%)
	Apr-Jun	16,849	246 (1.46%)
	Jul-Sep	13,511	169 (1.25%)
Total		40,336	593 (1.47%)

*missing ages of 6 VMMC clients

**Fig. 2:**
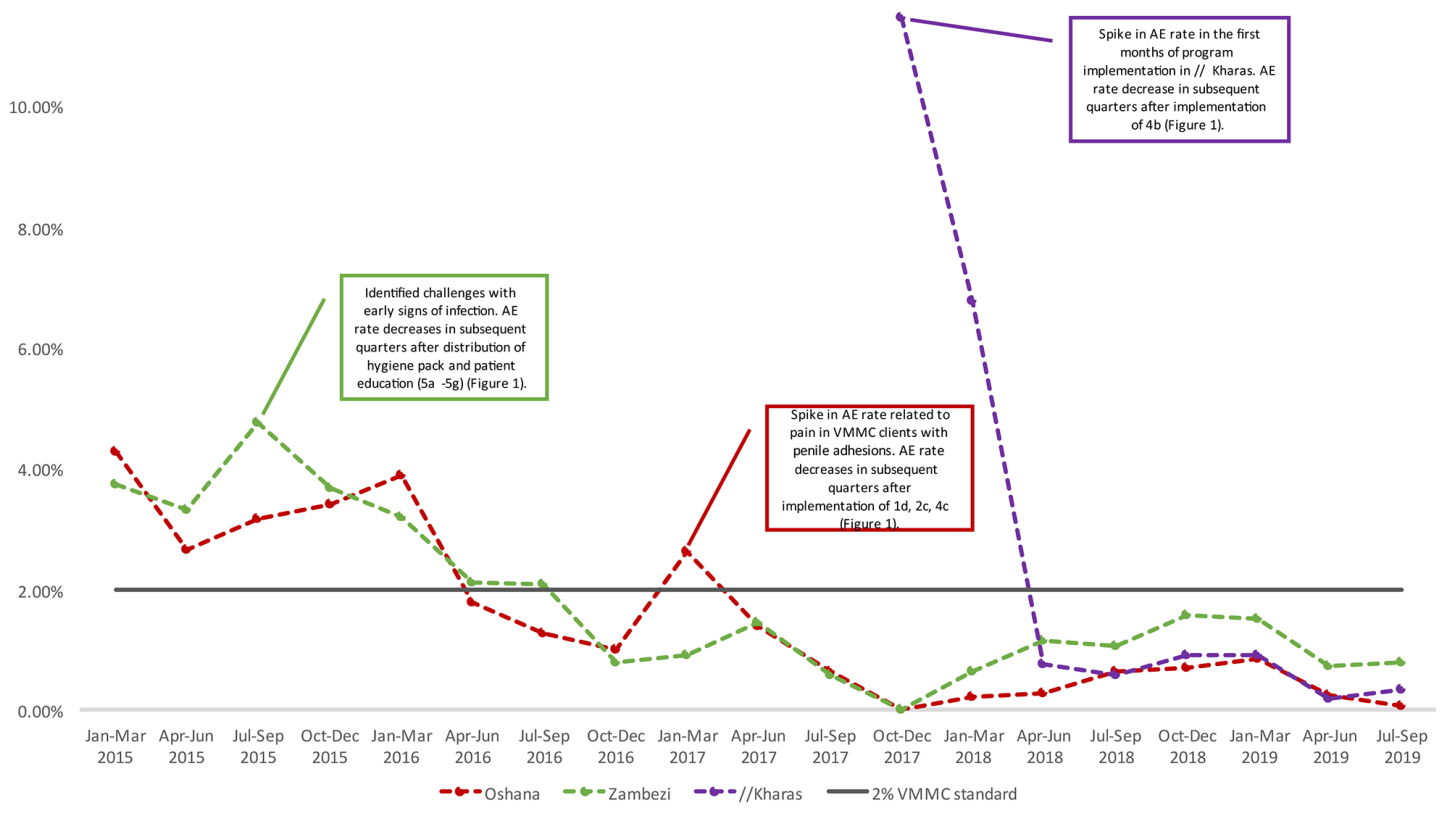
AE Rates by Quarter in the Oshana, Zambezi, and //Kharas regions in Namibia (January 2015–September 2019)

The AE rate was highest in the first quarter of clinical service delivery January-March 2015 in Oshana and Zambezi at 4.29 and 4.02 per 100 circumcisions performed and during the inception quarter of October-December 2017 in //Kharas at 11.49 per 100 circumcisions performed but subsequently consistently declined with increasing program maturation (Fig. 2). This observed trend between program maturity and declining AE rates over time was significant (p < 0.001) when compared using a Cochran-Armitage test for trend (Table 2).

**Table 2:** VMMC adverse event rate over time in the Oshana and Zambezi regions in Namibia (January 2015–September 2019)

		Total VMMC (N = 36,573)	Total AE (N = 547)	Proportion of VMMCs resulting in AE (95% CI*)	Chi2 for trend
Total	Year 1 (Jan 2015-Sep 2015)	6,251	207	3.31% (2.90–3.78)	< 0.001
	Year 2 (Oct 2015-Sep 2016)	7,686	166	2.16% (1.86–2.51)	
	Year 3 (Oct 2016-Sep 2017)	8,004	89	1.11% (0.90–1.37)	
	Year 4 (Oct 2017-Sep 2018)	7,592	41	0.54% (0.40–0.73)	
	Year 5(Oct 2018-Sep 2019)	7,040	44	0.63% (0.47–0.84)	
Oshana	Year 1	4,071	121	2.97% (2.50–3.54)	< 0.001
	Year 2	4,484	88	1.96% (1.60–2.41)	
	Year 3	4,619	54	1.17% (0.90–1.52)	
	Year 4	5,009	18	0.36% (0.23–0.57)	
	Year 5	3,828	13	0.34% (0.20–0.58)	
Zambezi	Year 1	2,180	86	3.94% (3.21–4.85)	< 0.001
	Year 2	3,202	78	2.44% (1.96–3.03)	
	Year 3	3,385	35	1.03% (0.74–1.43)	
	Year 4	2,583	23	0.89% (0.59–1.33)	
	Year 5	3,212	31	0.97% (0.68–1.37)	

*Wilson estimate of 95% CI

Three examples of locally and contextually developed quality initiatives are elaborated below. All relied on the expert knowledge of the context and community by the site teams complemented by a responsive team of experts and technical advisors to achieve success.

In Oshana, an identified spike in AE rates through review of the AE line list during a monthly technical and program feedback session (Fig. 1, 1d) led the team investigate an issue with penile adhesions between February and March 2017 (Fig. 2). At that time, the VMMC program was seeing many adolescent boys with penile adhesions. Penile adhesions can often be safely separated prior to the VMMC procedure, however clients reported that the separated adhesions resulted in increased pain, especially for school-aged clients who daily walk long distances to school. VMMC clients who experienced pain serious enough to result in at least one day of loss of work/normal activity (such as school) due to pain were classified as having a moderate AE [12]. After reviewing data and investigating underlying contributors to AEs, the QM team worked with the Physician Clinical Mentor to provide refresher trainings on clinical standard operating procedures (SOPs) including pain management (Fig. 1, 2c and 4c). Following implementation of these measures, the program observed a decrease in AE rates.

In Zambezi, teams identified poor hygienic conditions as a risk for AE and noted a pattern in increased AEs that could potentially have been attributed to poor hygiene (Fig. 2). In September 2015, the VMMC program team began distributing a hygiene pack containing a water bottle filled with one dose of pre-mixed saline to clients to facilitate proper post-surgical wound care, asking clients to purchase or find additional wound care supplies, specifically clean cotton wool and table salt to mix additional saline solution (Fig. 1, 5c). During follow-up, clinicians noted continued challenges with early signs of infection due to poor hygiene. Investigation by the program team identified an inability to afford and/or access supplies in many communities. As a result, the program began distributing a hygiene pack containing a water bottle, a bag of salt, and a bag of cotton balls (Fig. 1, 5e). It was further noted by clinicians that clients ran out of the distributed salt more quickly than anticipated because they were adding large and inconsistent amounts of salt when mixing additional saline solution. To mitigate this challenge, measuring spoons were added to the distribution package with guidance for correct mixing of the saline solution (Fig. 1, 5d). The Zambezi program team monitored a rapid decrease in AE rates after addressing challenges in maintaining post-surgical hygiene (Fig. 2). The distribution of wound care materials was adopted as a best practice, was subsequently instituted by the Oshana program, and became a key component of initial program implementation in the //Kharas VMMC site.

As the team initiated VMMC services in //Kharas from October 2017, the first months of service delivery monitoring identified an AE spike of 11.5% (Fig. 2). Recognizing the limited experience of this new team, a swift transfer of a senior VMMC clinician from an established site to the expansion site was implemented. This clinician had extensive clinical and community engagement and advocacy skills and conducted a VMMC IQA across the VMMC service continuum (including patient and community education and counselling and clinician mentoring and skills building) to identify and address quality gaps resulting in local team actions that reduced the AE rate to below the 2% international benchmark of acceptability within 6 months (Fig. 1, 4b and Fig. 2).

## Discussion

As the I-TECH VMMC program matured, successive QM measures were introduced and routinized. The program observed a significant decrease in AE rate over the course of the implementation period aligning with introduction of QM measures suggesting that clinical quality improved over time. These results are in line with previous studies suggesting the linkage between program duration and improved program quality. Maturity models or capability frameworks have established that the maturity of healthcare program processes, practices, and infrastructure can improve efficiency of service delivery [32]. A multi-country analysis of facilities providing VMMC in sub-Saharan Africa also found that sites implementing for longer periods of time, and therefore considered more mature, fell into higher quality quadrants [33]. Stepwise review of data and implementation of QM interventions appear effective in assuring patient safety over time. Several lessons learned, however, merit discussion.

Few studies explore the influence of QM activities within routine service delivery. In South Africa, a set of continuous quality improvement (CQI) action plans were applied to guide focused coaching, application of guidelines, and on-site training to improve routine VMMC program quality across the spectrum of VMMC program delivery finding that average program quality increased significantly (18% (95%CI: 14–21; p < 0.001) across CQI categories with larger gains in management and M&E over those observed in VMMC counselling or surgical skills [34]. A previous multi-country process evaluation in Eastern and Southern Africa found substantial differences by site and over time, but that overall efficiency gains (defined as decreased provider time with client and elapsed operating time) did not result in decreased quality [4, 35]. An analysis of a large scale VMMC program in Tanzania found a declining AE rate over time thought to be attributable to a focus on systems thinking to prevent and report AEs [36]. Together, these findings suggest that comprehensive QM interventions are needed to expand beyond surgical oversight and AE monitoring to include a systematic approach to improving quality across the continuum of VMMC service delivery from patient and community education and counselling to infection prevention and control, pre-operative examinations, intra-operative surgical procedures, post-operative care, clinical management support systems, and M&E.

There were several key concrete QM steps implemented as part of our program that VMMC or service delivery teams elsewhere may be able to implement. Starting in Oshana and Zambezi, and later in //Kharas, a multi-disciplinary team reviewed and discussed program data and AE rates on a weekly basis. Above-site technical advisors also made frequent in-person support visits to provide supportive supervision and mentorship and coaching. During those data reviews and site visits, conversations were held with the clinicians and clients and operations were observed to identify variations in quality standards. These reviews led to data-driven identification of problems, promoted joint team generation of possible solutions, and suggested specific new interventions for testing in support of continuous quality improvement.

### Limitations

This study and findings are subject to several limitations. First, although implemented in three regions, overall absolute eligible target population numbers for VMMC are small, allowing for integration of intensive QM efforts using dedicated staffing and resources. The program had adequate technical assistance to step back from implementation and review data, identify solutions, and test change. This level of QM effort and resource application would likely not be sustainable on a national scale in less highly resourced regions. Second, these QM efforts were successful for the VMMC program but had limited direct benefit to the larger health systems in which the VMMC program was implemented. Future efforts should ensure that QM gains in a specific program will support horizontal approaches that strengthen other priority programs and the larger health system through capacity building, empowering local ownership, and developing cross-cutting quality management infrastructure [37]. Third, this analysis only included AEs that were classified as moderate and severe by clinicians, those classified as minor AEs are not included. Fourth, although QM efforts included activities to improve the consistency in reporting and classifying AEs, variation existed across sites, regions, and clinicians during the implementation period. Fifth, although follow-up completion was near 100%, it is possible that a small number of VMMCs resulting in AEs were missed due to incomplete follow-up. Rates of AEs were calculated using all VMMCs as the denominator, not excluding VMMC without complete follow-up, because the number of VMMCs with incomplete follow-up was so small. Sixth, this was a facility based VMMC program and only included VMMCs performed at static and outreach VMMC sites served by healthcare providers in regions without a culture of traditional circumcision. Future research should explore patterns of VMMC and rates of AEs in traditional circumcision.

## Conclusions

Findings from this study lead to several programmatic recommendations that may further improve clinical service quality in Namibia’s VMMC program and are also applicable to other VMMC programs. Key findings and recommendations from this analysis include: (1) routinizing QM processes as the program matured aligned with an observed increase in clinical safety and improvement in program outcomes, (2) QM processes and approaches were applied across the continuum of VMMC services including client and community facing education and counselling, (3) QM efforts carefully considered and responded to the local context and were implemented using a multi-disciplinary team approach spanning multiple levels of the health system, (4) QM measures and experience established in more mature program sites can be applied to rapidly and efficiently resolve issues arising in program expansion sites with care taken to continue to be responsive to the specific sub-population and community needs in the expansion site. Patient safety and clinical outcomes are critical as VMMC programs continue to scale to meet ambitious national and international targets.

## Data Availability

All data analyzed for this study are included in this published article.
